# Direct oral anticoagulants and venous malformations: literature review and retrospective study of 29 patients

**DOI:** 10.1016/j.rpth.2024.102400

**Published:** 2024-04-03

**Authors:** Eugénie Lagneaux, Laurence M. Boon, Nicole Revencu, Miikka Vikkula, Cedric Hermans

**Affiliations:** 1 Division of Adult Hematology, Cliniques Universitaires Saint-Luc, Université Catholique de Louvain (UCLouvain), Brussels, Belgium; 2 Center for Vascular Anomalies, Cliniques Universitaires Saint-Luc, Université Catholique de Louvain (UCLouvain), VASCERN VASCA European Reference Centre, Brussels, Belgium; 3 Division of Plastic Surgery, Cliniques Universitaires Saint-Luc, Université Catholique de Louvain (UCLouvain), VASCERN VASCA European Reference Centre, Brussels, Belgium; 4 Human Molecular Genetics, de Duve Institute, Université Catholique de Louvain, Brussels, Belgium; 5 Department of Clinical Genetics, Cliniques Universitaires Saint-Luc, Université Catholique de Louvain (UCLouvain), VASCERN VASCA European Reference Centre, Brussels, Belgium

**Keywords:** D-dimer, direct oral anticoagulant, localized intravascular coagulopathy, pain, venous malformation

## Abstract

**Background:**

Venous malformations (VMs) are commonly associated with localized intravascular coagulopathy leading to elevated D-dimer and risks of hemorrhagic and thromboembolic events, particularly in extensive lesions. While low-molecular-weight heparin (LMWH) has been effective in managing coagulopathy and pain, direct oral anticoagulants (DOACs) emerge as a promising alternative.

**Objectives:**

This study aims to evaluate the efficacy and safety of DOACs in treating VMs associated with localized intravascular coagulopathy, offering a comparative perspective to LMWH.

**Methods:**

A retrospective study was conducted on 29 patients with VMs and secondary localized intravascular coagulopathy treated with DOACs between 2013 and 2023 in a single tertiary center specialized in vascular anomalies. Data were collected from February 24, 2023, to September 1, 2023.

**Results:**

Patients’ median age was 40 years (range, 22-76 years), with a female predominance of 66%. Descriptive statistical analysis showed that 85% of patients experienced pain improvement, and 86% showed a reduction in D-dimer by at least 25%, with a mean reduction of 57% (SD, ±32%; IQR, [38-81%]). Additionally, 37% of patients reported a bleeding event, mostly minor.

**Conclusion:**

The study findings suggests that DOACs may serve as an alternative to LMWH for patients with VMs associated with pain management and reduced D-dimer, alongside a low observed risk of major bleeding. Tailored dosing considering the location of the malformation, bleeding and thrombotic tendencies, and laboratory abnormalities is recommended. Future studies with larger cohorts and extended follow-up are necessary for more conclusive evidence on DOACs’ role in this patient population.

## Essentials

• Venous malformations (VMs) often lead to clotting, bleeding, and pain.
• Twenty nine patients with VMs were treated with direct oral anticoagulants.
• Reduced pain and D-dimer were observed in most patients, with no major bleeding.
• Direct oral anticoagulants offer a potential alternative to low-molecular-weight heparin in patients with VMs.

## Graphical abstract

**Figure:**
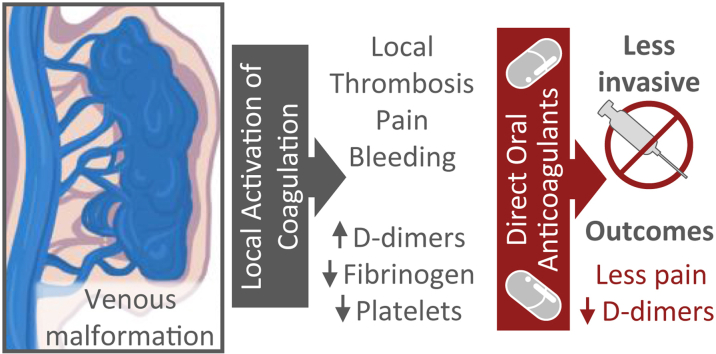

## 1 Introduction

Venous malformations (VMs) are slow-flow vascular malformations that occur due to developmental errors in the venous network, resulting in dilated and dysfunctional veins. They are present at birth and grow proportionally with age, affecting 1 in 2000 births. They are the most frequent vascular malformation seen in specialized centers [1,2]. VMs are caused by somatic mutations in genes involved in blood vessel development and maintenance, such as *TEK* and *PIK3CA* in 60% and 20% of cases, respectively [[3], [4], [5]].

VMs typically appear as bluish or purple lesions primarily in the skin, the mucosa, or the subcutaneous tissue, but they can affect any tissue or organ. While most VMs are unifocal, some present as multifocal. These multifocal VMs can be associated with lymphatic or capillary malformations. Additionally, they may be linked to specific clinical syndromes or characteristics. For instance, they can be part of the blue rubber bleb nevus syndrome (OMIM %112200) or the capillary malformation with dilated veins. Moreover, they might be a component of broader syndromes such as the Klippel–Trenaunay syndrome (OMIM %149000) or the congenital lipomatous overgrowth, vascular malformations, epidermal nevi syndrome (OMIM # 612918) [6]. All these entities manifest as vascular malformations, but they present uniquely distinguishing features.

Symptoms vary by lesion size and location causing swelling, pain, bleeding, disfigurement, and functional impairment, often exacerbated during menstruation or physical activity. Intra-articular VMs can lead to pseudohemophilic arthropathy, characterized by joint pain, stiffness, and progressive destruction of the joint cartilage and bone because of repeated episodes of joint bleeding [7,8]. Early surgery in patients with intra-articular VM, even if asymptomatic, is important to prevent joint impairment [9].

The deficiency of vascular smooth muscle cells in these venous lakes leads to blood stagnation, activation of the coagulation cascade, local thrombus formation, and subsequent breakdown, consuming clotting factors and increasing D-dimer. This condition, known as localized intravascular coagulopathy or consumptive coagulopathy, occurs in approximately 40% of patients with VMs. It is associated with a higher risk of diffuse intravascular coagulation (DIC), which can lead to severe bleeding during surgical procedures [10]. Risk factors for DIC include sclerotherapy, surgery, bone fracture, immobilization, pregnancy, and sepsis [10]. Therefore, evaluating blood coagulation parameters before interventions and initiating preventive anticoagulation therapy in cases of localized intravascular coagulopathy is essential [11]. Patients with extensive VMs face a higher risk of thromboembolic events, such as deep venous thrombosis (DVT) and pulmonary embolism (PE), compared with those with localized VMs [[10], [11], [12], [13]].

The primary goals of VM treatment are to reduce pain and functional impairment and prevent bleeding and thrombotic complications. Therapeutic options include compression garments, painkillers, sclerotherapy, endovascular laser treatment, surgical excision, and anticoagulation therapy for individuals with underlying coagulopathy [2,6,14]. Sirolimus, a theranostic medication, has proven to be efficacious in 85% of cases and is now widely used [15,16].

Low-molecular-weight heparin (LMWH) is the only anticoagulant therapy known to improve pain, prevent bleeding and thrombotic complications, and treat thrombotic events in VMs [6,10,11,13,14]. LMWH is used in case of pain with elevation of D-dimer, in thrombotic events (acute phase and long-term secondary prophylaxis), in primary prophylaxis of thrombotic and bleeding events in case of decreased fibrinogen levels, or before sclerotherapy or surgery for patients with elevated D-dimers or large VMs. Although effective, LMWH has limitations, such as subcutaneous injections and potential side effects like heparin-induced thrombocytopenia and osteoporosis after prolonged use.

Direct oral anticoagulants (DOACs) are being explored as a promising alternative to LMWH. DOACs directly inhibit key enzymes in the coagulation cascade, either thrombin or factor Xa, and can potentially improve pain and reduce thrombotic and hemorrhagic risk by controlling localized intravascular coagulation. DOACs offer numerous advantages such as no monitoring requirement, rapid reversibility, multiple dosages, minimal interactions, and no need for injections [17].

To date, there have been 46 reported cases in the literature of patients using DOACs in the context of localized intravascular coagulopathy associated with VMs [[18], [19], [20], [21], [22], [23], [24], [25], [26], [27]] (Supplementary Table S1). The largest case series concerns 19 patients taking dabigatran [27]. Hereunder, we present what we believe to be the largest case series of 29 patients with VMs treated with 3 types of DOACs—rivaroxaban, apixaban, and dabigatran—in our center.

## 2 Methods

We conducted a retrospective cohort study at our tertiary multidisciplinary center for vascular anomalies, analyzing medical records from 2013 to 2023. The study focused on patients with varying degrees of VMs and secondary localized intravascular coagulopathy who were prescribed DOACs. These patients either started DOACs as their initial antithrombotic treatment or switched from LMWH or acenocoumarol to DOACs. They were referred to the Hemostasis and Thrombosis Unit for assessment, DOAC initiation, and follow-up.

The study was approved by the local ethical committee (approval reference 2023/24FEV/101). Data were collected from February 24, 2023, to September 1, 2023. The patients provided oral informed consent and underwent biologic blood testing and routine examinations before and after treatment.

**Figure 1:**
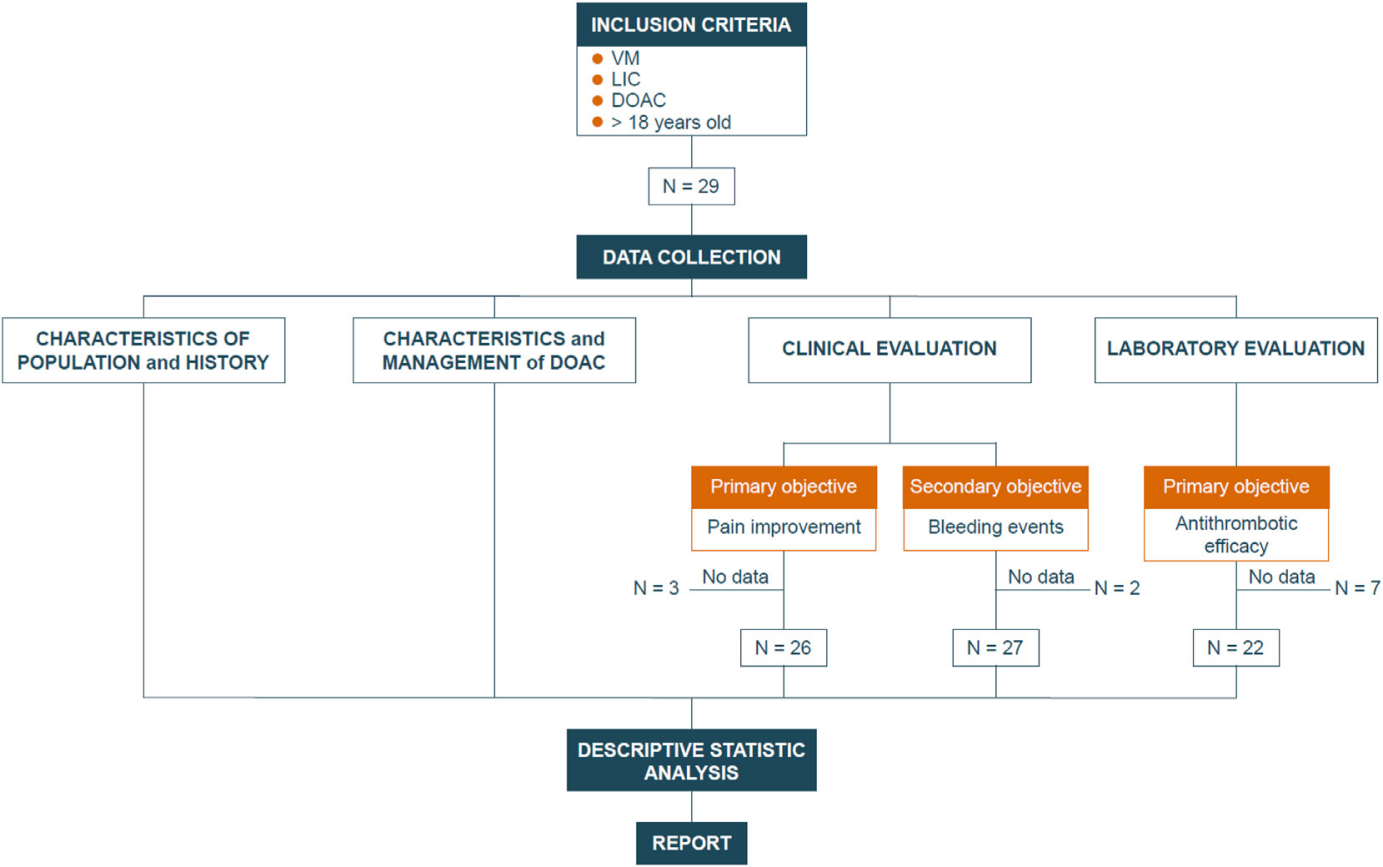
Flowchart of the study. The retrospective evaluation of direct oral anticoagulants (DOACs) in 29 adult patients with venous malformations (VMs) and localized intravascular coagulopathy (LIC). It outlines the study’s inclusion criteria, dual clinical and laboratory assessments of therapy effectiveness, and the statistical analysis process.

The primary objectives were to evaluate the effectiveness of DOACs in reducing pain associated with VMs and to assess the antithrombotic efficacy of DOACs by measuring levels of D-dimer expressed in nanograms per milliliter, using fibrinogen equivalent units before and after treatment. Improvement in D-dimer was defined as a reduction of more than 25% in D-dimer values. The choice of a 25% reduction in D-dimer values as the cutoff for defining improvement was made arbitrarily due to the absence of a formal biological rationale in the literature. The secondary objective was to examine bleeding events under DOAC (Figure 1).

We conducted descriptive statistical analysis to investigate patient characteristics, management of DOACs, laboratory results, and clinical evaluation (Supplementary Table S2).

## 3 Results

### 3.1 Characteristics of study population

We analyzed data of a total of 29 patients with VMs treated with DOACs between 2013 and 2023 (Table). Supplementary Table S3 provides an overview of patient data and main results. The median age of these patients was 40 years with a range of 22 to 76 years, with 66% of them being women and girls. Extensive VMs, defined as affecting more than 9% of their body surface area, were observed in 76% of patients, with the lower leg being the most affected area in 59% of patients. Sixteen patients (55%) had an identified variant in *PIK3CA* (*N* = 8) or *TEK* (*N* = 8), while the 13 others have not been investigated.

**Table:** Clinical characteristics of study population.

VARIABLE	N
**Number of patients**	29
**Median age (y)**	40 (Range : 22 to 76 years)
**F/M**	19 (66%) / 10 (34%)
**Race**	Caucasian (27)
	African (2)
**Size of VMs**	Localized : 7 (24%)
	Extensive : 22 (76%)
**Disorders**	KT : 3
	BRBNS : 1
	CLOVES : 2
	CMDV : 1
	MSVM : 1
	Sporadic VM : 21
**Localisation of VMs**	Leg : 17 (59%)
	Whole body : 4 (14%)
	Trunk : 11 (38%)
	Perineal : 3 (10%)
	Arm : 7 (24%)
**Genetic mutation**	PIK3CA : 8 (28%)
	TEK : 8 (28%)
	N/A : 13 (45%)
**Previous anticoagulation**	Intermittent LMWH : 23 (79%)
	Continuous LMWH : 3 (10%)
	Acenocoumarol : 5 (17%)
	None : 3 (10%)
**Pseudo-hemophilic arthropathy**	8 (28%)
**Thromboembolic event**	
**SVT**	22 (76%)
**DVT**	6 (21%)
**PE**	3 (10%)
**DIC**	4 (14%)
**Postoperative haemorrhage**	5 (17%)

This table summarizes the demographics, clinical characteristics, prior anticoagulation, and history of thromboembolic events of 29 patients with venous malformations (VMs). It is important to note that some patients presented with more than one localization of VMs, were treated with multiple types of anticoagulation, and experienced various thromboembolic events, which may lead to the total counts exceeding 29 in certain categories. Notable abbreviations and terms include: F (Female), M (Male), KT (Klippel-Trenaunay Syndrome), BRBNS (Blue Rubber Bleb Nevus Syndrome), CLOVES (Congenital Lipomatous Overgrowth, Vascular Malformations, Epidermal Nevi, Skeletal Anomalies syndrome), CMDV (Capillary Malformation with dilated veins), MSVM (Multiple sporadic Venous Malformation), LMWH (Low Molecular Weight Heparin), SVT (Superficial Vein Thrombosis), DVT (Deep Vein Thrombosis), PE (Pulmonary Embolism), and DIC (Disseminated Intravascular Coagulation).

Twenty six out of 29 patients (90%) had received previous antithrombotic treatment such as intermittent or continuous LMWH or acenocoumarol, primarily for superficial venous thrombosis (SVT) (20 patients). Other indications included DVT, PE, pregnancy, or surgery/sclerotherapy. Eight patients had pseudohemophilic arthropathy, with knee involvement in 7 (alone in 4, combined with hip in 2, and with hip and ankle in 1).

Regarding thromboembolic events history before DOAC treatment, 22 patients (76%) reported at least 1 episode of SVT, 6 patients (21%) experienced at least 1 episode of DVT, and 3 patients (10%) had an episode of PE. Four patients (14%) experienced DIC in a postoperative setting. These episodes occurred after cesarean section, sclerotherapy, splenectomy, varicectomy, or inguinal hernia surgery and were accompanied by severe bleeding.

Out of the 6 patients screened for bleeding disorders, 1 had mild hemophilia, took apixaban but discontinued after a month due to inefficacy and bleeding concerns. Among the 9 patients screened for thrombophilia, one had a prothrombin G20210A mutation, and another had a factor V Leiden mutation. Interestingly, neither of these 2 patients experienced DVT or PE, but all did have SVT.

### 3.2 DOAC management

The decision to initiate DOAC treatment was multifactorial, driven by various reasons including, a history of SVT or DVT/PE, or pain with elevated D-dimer. One patient started DOAC treatment based on laboratory criteria of low platelet count, low fibrinogen levels, and high D-dimer, despite no pain or thromboembolic incidents, aiming to mitigate hemorrhagic or thrombotic complications through managing consumptive coagulopathy.

Among the DOACs used, apixaban was the most preferred choice, prescribed to 18 patients (62%), followed by rivaroxaban, which was chosen for 7 patients (24%) who preferred once-daily intake. Dabigatran was intentionally prescribed to 8 patients (28%) with VMs associated with a high risk of life-threatening bleeding owing to the location of the malformation and the availability of a specific antidote (idarucizumab, Praxbind, Boehringer Ingelheim). Four patients (14%) were sequentially treated with 2 different types of DOACs due to thrombotic event, or reimbursement issues (Supplementary Table S3).

To mitigate bleeding risks and account for the limited data available on this specific indication, we adopted a gradual approach to increase the anticoagulant dose based on individual patient tolerability, bleeding symptoms, and D-dimer. Initially, low doses of anticoagulants such as apixaban 2.5 mg twice daily, rivaroxaban 10 mg once daily, or dabigatran 75 mg twice daily were administered, and patients were re-evaluated after 1 to 3 months. If the response was deemed insufficient (persistent pain, thrombotic event, or persistent elevated D-dimer), the dose was increased to an intermediate (rivaroxaban 15 mg once daily or dabigatran 110 mg twice daily) or high dose (rivaroxaban 20 mg once daily, apixaban 5 mg twice daily, or dabigatran 150 mg twice daily) of the respective DOAC. Six patients required an increase in dosage: 2 because of ongoing pain and high D-dimer after 1 and 2 months of treatment, respectively; 2 because of the onset of SVT after 48 and 13 months; 1 for persistently elevated D-dimer (over 35,000 pg/mL) after 3 months; and the last 1 following a PE after 76 months of treatment. Three patients had to switch to a very low dose due to gynecologic, urological bleeding and hematochezia. Within this group, 2 patients experienced a thrombotic event—one with SVT and another with a PE—under the very low dose, necessitating a subsequent increase in dosage. Patient 9 (Supplementary Table S3) had multiple VMs and 3 consecutive PEs. The first PE led to acenocoumarol initiation, but subsequent PEs occurred despite subtherapeutic doses. Following a switch to a lower dose of rivaroxaban (15 mg) due to menorrhagia, the dose was subsequently increased to 20 mg after menopause due to a third PE episode.

Eight patients were switched from heparin (*n* = 5) and acenocoumarol (*n* = 3) to DOACs. For most patients, we also initiated treatment with low dose of anticoagulant, incrementally increasing it as necessary. However, for some patients, including patient 17, who struggled with significant pain and management issues on well-dosed acenocoumarol, and patient 22, with recurrent thromboembolic disease, we started with higher doses. An exception was made for patient 9, who had recurrent thromboembolic event and should have been on a high dose, but due to menorrhagia, we initiated treatment with an intermediate dose. Consequently, among the 8 patients already on anticoagulants, 5 commenced with lower doses, 1 with an intermediate dose, and 2 with higher doses.

Finally, 16 patients (55%) were on low-dose DOACs, 3 patients (10%) were on intermediate-dose DOACs, and 9 patients (31%) were on high-dose DOACs. One patient received a very low dose of DOAC (apixaban 2.5 mg once daily) due to recurrent rectal bleeding caused by the perineal location of the malformation. The average duration of DOAC treatment was 3.4 years, ranging from 1 month to 10 years.

### 3.3 Anticoagulation management of surgery and pregnancy

Fourteen interventions were performed while patients were on DOAC therapy: 1 ocular surgery, 3 arthroplasties, 1 appendicectomy, 6 varicectomies, and 3 sclerotherapies. Most of these interventions were performed without bridging anticoagulation. For patients on low-dose DOACs, the last dose was taken on the morning of the day before surgery (D−1) or 2 days before (D−2) in case of surgery with high bleeding risk [28]. For patients on high-dose DOACs, the last dose was taken 2 days before surgery (D−2). Semitherapeutic doses of LMWH were resumed 6 to 8 hours after surgery, followed by therapeutic doses on the first day after surgery (D+1). DOAC therapy was resumed between 2 and 7 days after surgery (D+2 to D+7) (Supplementary Figure S1).

One pregnancy was reported. The patient had to discontinue DOAC and switch to LMWH. Regular follow-up appointments were scheduled every 6 weeks during pregnancy, during which targeted prophylactic anti-Xa dosages were measured. For delivery, the last dose of LMWH was administered on the morning of the day before delivery (D−1), and LMWH therapy was resumed 6 to 8 hours after delivery.

### 3.4 Laboratory tests

Laboratory tests revealed that 14 out of 29 patients (48%) had maximal D-dimer exceeding 10,000 ng/mL, indicating significant activation of the coagulation cascade. D-dimer were assessed before and after treatment for 22 out of 29 patients, as 2 patients were lost for follow-up, 3 had recently initiated treatment, and 2 stopped the treatment before because of inefficiency (*n* = 1) or pregnancy (*n* = 1). The median time of measurement of D-dimer after starting treatment was 4.9 months (ranging from 1 to 96 months).

**Figure 2:**
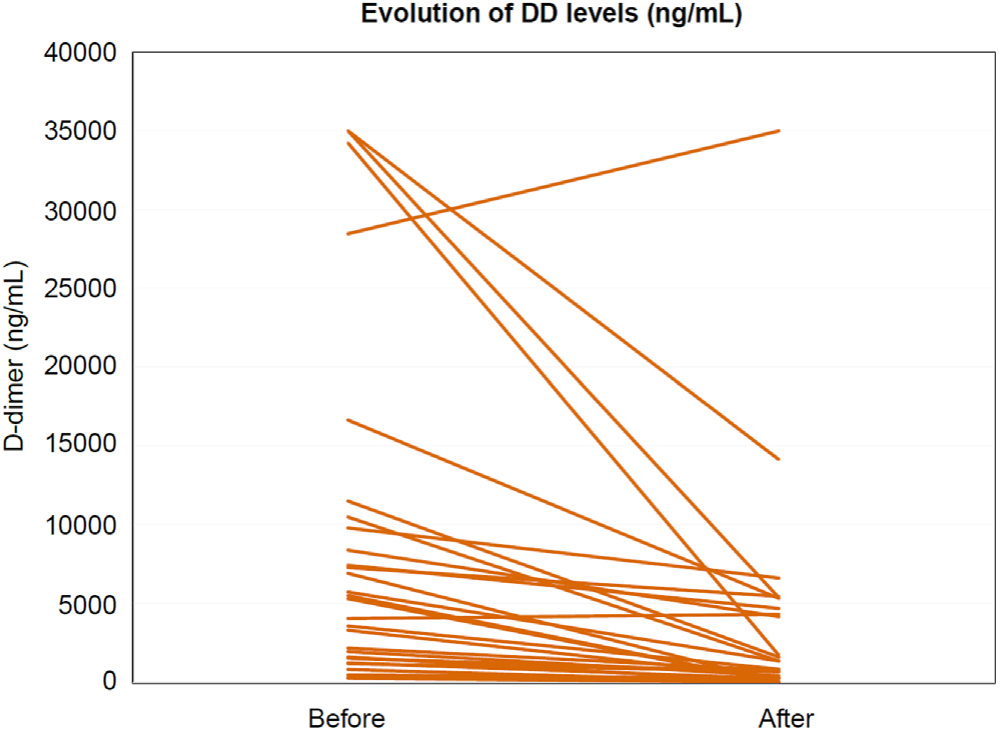
D-dimer (DD) before and after direct oral anticoagulant (DOAC) therapy. This graph displays the change in DD (expressed in nanograms per milliliter, using fibrinogen equivalent units) in patients before and after the initiation of DOACs therapy. The median time to follow-up measurement was 4.9 months, with a range from 1 to 96 months. The mean DD prior to treatment was 8376 ng/mL (SD, ±10,885 ng/mL; IQR, [1210-9787 ng/mL]), which reduced to 4157 ng/mL (SD, ±7645 ng/mL; IQR, [465-5162 ng/mL]) after treatment with DOACs, indicating a significant decrease. Although the considerable mean reduction indicates a potentially strong effect of the treatment, the high SDs and IQRs point to substantial variability among individual responses.

Among the evaluated patients, D-dimer decreased by at least 25% in 19 out the 22 evaluable patients (86%) and by at least 50% in 15 patients (68%). The study observed a significant reduction in D-dimer following DOAC therapy, with the mean levels decreasing from 8376 ng/mL (SD, ±10,885 ng/mL; IQR, [1210-9787 ng/mL]) before treatment to 4157 ng/mL (SD, ±7645 ng/mL; IQR, [465-5162 ng/mL]) after treatment. The mean and median reduction percentages in D-dimer were 57% (SD, ±32%; IQR, [38-81%]) and 67%, respectively (Figure 2). Although the considerable mean reduction indicates a potentially strong effect of the treatment, the high SDs and IQRs point to substantial variability among individual responses. During the assessment, it was observed that 5 patients (17%) had a platelet count nadir lower than 100,000/microliter, and 8 patients (28%) had a fibrinogen level nadir lower than 150 mg/dL.

When we examined the reduction in D-dimer percentage for each treatment dose category (very low, low, intermediate, and high) (Supplementary Figure S2), we noticed that responses appeared to be more favorable with lower doses. It is important to note that this observation does not necessarily indicate lower effectiveness of higher doses. Instead, it suggests that patients benefiting from higher-dose anticoagulation for managing refractory pain and/or thromboembolic episodes likely have more severe underlying conditions, making coagulation control more challenging and, consequently, resulting in a lesser decrease in D-dimer. Therefore, the apparent trend should be understood in the context of disease severity, which influences both the dosing strategy and the resulting treatment outcomes.

### 3.5 Clinical evaluation

**Figure 3:**
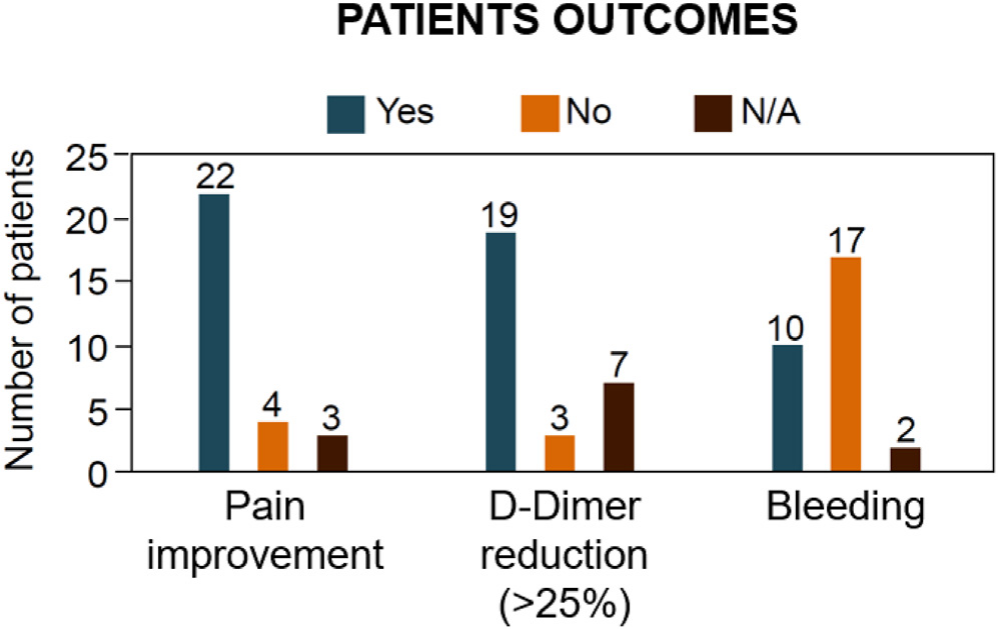
Patient outcomes following direct oral anticoagulant therapy. This bar chart presents data on patients treated with direct oral anticoagulants, documenting instances of pain relief, reductions in D-dimer (defined as a decrease of more than 25%), and bleeding occurrences. Responses are categorized as “Yes” for improvement or event observed, “No” for no improvement or event absence, and “N/A” for unavailable data or nonapplicability. It is noted that a majority reported pain relief and a significant reduction in D-dimer, while bleeding events were less commonly reported. These data reflect a potentially favorable risk-benefit profile for direct oral anticoagulants in this cohort.

A total of 22 (85%) patients demonstrated a pain reduction, while 4 (15%) patients did not show any improvement. Three patients were not included in the evaluation: 2 were lost to follow-up, 1 did not experience pain at the beginning of the treatment (Figure 3).

When analyzing the 4 nonresponders, it becomes evident that all of them have extensive malformations. Additionally, 3 of these nonresponders are the same individuals who had a history of PE, indicating the presence of an underlying severe coagulopathy. Nevertheless, it is worth noting that 2 of these 4 nonresponders did demonstrate an improvement in D-dimer. Gender did not significantly affect the results, as half of the nonresponders were male, and half were female.

In the evaluation of pain relief across different therapeutic dose categories—ranging from very low to high—it has been observed that lower doses are associated with a higher percentage of positive outcomes, as detailed in Supplementary Table S4. This pattern is similar to that noted for D-dimer reductions. Patients presenting with more severe clinical presentations are often prescribed higher doses. This observation suggests that the severity of the condition may influence both the dose administered and the subsequent response to therapy, with more challenging cases potentially requiring higher dosages for effective coagulation control.

Among the 2 patients who underwent a dose increase due to uncontrolled pain at 1 and 2 months after starting treatment, the increased dosage successfully relieved pain in one of them. In contrast, for the 2 patients who had their dosage increased following a PE episode, this adjustment did not result in any improvement in their pain symptoms.

It was not possible to establish a correlation between the reduction in D-dimer and the improvement in pain symptoms. However, it is worth noting that among the patients with pain improvement (*n* = 22), only 1 patient did not show an improvement in D-dimer.

Regarding bleeding events during DOAC therapy, 10 patients (37%) reported at least 1 bleeding event, which were predominantly minor. These events included hematuria (*n* = 1), gingival bleeding (*n* = 2), epistaxis (*n* = 1), hematochezia (related to the anorectal location of the vascular malformation) (*n* = 4), and menorrhagia (*n* = 4). One patient experienced joint bleeding secondary to pseudohemophilic arthropathy. Seventeen patients (63%) did not report any bleeding events. Information on bleeding events was missing for 2 patients due to loss to follow-up.

Six patients experienced thromboembolic event while under treatment, including 1 cases of SVT while on apixaban 2.5 mg once daily, 1 case of SVT while on apixaban 2.5 mg twice daily, 2 cases of SVT while on dabigatran 110 mg twice daily, 1 case of PE while on rivaroxaban 15 mg once daily, and 1 case of postarthroplasty PE while on rivaroxaban 10 mg once daily. Only 1 case of DIC was detected in a postoperative context following varicectomy in a patient who did not show any improvement in D-dimer on dabigatran 110 mg twice daily (>35,000 ng/mL). This demonstrates that DOACs, while effective given the reduction in the number of thrombotic events typically expected in this population, do not provide absolute prevention, particularly with low dosages, or in postoperative state.

## 4 Discussion

This study represents, to our knowledge, the largest case series using DOACs in VM management, particularly focusing on pain management and antithrombotic properties. Apixaban was the predominant choice, followed by rivaroxaban for those prioritizing once-daily administration and finally dabigatran for patients with VMs prone to hemorrhagic manifestations. We titrated anticoagulant dosages based on tolerance, hemorrhagic and thrombotic manifestations, pain, and D-dimer. Most participants (85% and 86%, respectively) observed marked pain relief and a decrease in D-dimer by over 25% during DOAC therapy, without major bleeding episodes.

Comparing our results with the study by Liu et al. [27], which included 19 patients treated with dabigatran, our outcomes in terms of pain reduction were similar (84%). Regarding the decrease in D-dimer, they reported a rate of improvement of 95%. The discrepancy could be attributed to our decision to use a 25% reduction as a significant benchmark despite the lack of a formal biological rationale, as most studies do not specify a cutoff. Without such a cutoff, our rate of improvement in D-dimer closely matches theirs at 91%.

Our findings show a mean D-dimer reduction of 57% (SD, ±32%; IQR, [38-81%]) with DOAC use, slightly below the 62.8% seen with LMWH in the 22-patient study by Dompmartin et al. [11]. The timing of D-dimer measurements differed significantly, with ours extending over several months vs 1 month in the research by Dompmartin et al. [11]. Dompmartin et al. [11] reported pain improvement in all patients. The difference can be explained by dissimilar patient populations. In the study by Dompmartin et al. [11], patients with involvement in more than 2 regions were excluded. Most participants, accounting for 92%, had only 1 region affected, whereas 6% had 2 regions involved. In contrast, our study included a population with more severe disease, as 48.3% of our patients had only 1 affected region, while 20.7% exhibited involvement in more than 2 regions. This distinction suggests that our population may have been more medically compromised, potentially making them more refractory to treatment. Patients in our practice experienced similar pain relief with LMWH and DOACs, preferring DOACs for their injection-free convenience and greater satisfaction.

An unexpected finding was the distribution of TEK and PIK3CA genetic variants. Contrary to the dominant literature reporting about 60% for TEK variants and 20% for PIK3CA, the study found an equal 50% distribution for each. Furthermore, when assessing pain improvement based on genetic variants, all TEK variant carriers experienced relief in pain symptoms, while 25% of PIK3CA carriers did not. It is also essential to note that 2 out of the 4 nonresponders in the study had PIK3CA variants. This suggests that PIK3CA variant carriers may have a more severe condition and are less responsive to anticoagulant therapy, although the small sample size (8 patients per variant group) necessitates cautious interpretation.

Sirolimus was administered to 20 of the 29 patients either before, during, or after DOAC therapy, which could serve as a confounding factor in assessing efficacy. Eleven patients discontinued sirolimus before DOAC therapy because of intolerance (*n* = 7) and ineffectiveness (*n* = 4). To date, no synergistic effect between these medications has been demonstrated. Both DOACs and sirolimus are substrates for CYP3A4 and P-glycoprotein, suggesting potential competition in their metabolic pathways. Although sirolimus is known to mildly inhibit CYP3A4, existing studies have not indicated a significant impact on DOAC levels [29], and no dosage adjustments were made for these patients.

In our study, all patients with localized malformations found pain relief with anticoagulation, unlike those with extensive malformations, particularly if they had a history of PE. Higher doses of anticoagulants did not always mean better pain control, possibly due to the more severe disease in these patients. This suggests 2 key points: anticoagulation can help with pain in localized malformations, irrespective of their thrombosis history, but for extensive cases, especially those with past PE, higher doses may not improve pain relief.

Despite its retrospective, single-center and noncontrolled design, this study’s strengths lie in its large sample size and the variety of DOAC types and dosages used. The results suggest DOACs as a viable alternative to LMWH for managing pain and localized intravascular coagulopathy in patients with VMs owing to their convenience, less invasive administration, and easy and safe perioperative management. Low doses of anticoagulants could be a feasible option for significant pain improvement in many patients. The risk-benefit ratio should be assessed individually, and the dose must be tailored for each patient based on the location of VM, bleeding tendency, and laboratory monitoring. Rigorous clinical and laboratory surveillance is imperative to ensure their safe and effective use.

Exploring the feasibility of employing DOACs among patients with VMs lacking pain and thrombotic manifestations but with elevated D-dimer, outside of the perioperative context, presents a compelling avenue. Could this approach mitigate thrombosis, hemorrhage, or sudden death risks within this subset? This inquiry sparks a discussion deserving thorough exploration, potentially reshaping therapeutic strategies for these patients.

## 5 Conclusion

DOACs offer a promising alternative to LMWH for long-term antithrombotic treatment of patients with VMs and localized intravascular coagulopathy. Study findings indicate an association between DOAC use and improved pain control and D-dimer reduction, with a minimal observed risk of significant bleeding, potentially leading to an improvement in quality of life for individuals with VMs.

## Supplementary material

Figure S1 **Perioperative Management of Anticoagulation in Patients on direct oral anticoagulant** The timeline details anticoagulation management for patients undergoing surgery. For low-dose DOAC patients, the last dose was administered either the morning before surgery (D-1) or two mornings prior for surgeries with a high risk of bleeding (D-2). High-dose DOAC patients stopped their medication two days before surgery (D-2). Postoperatively, semi-therapeutic doses of LMWH were started 6-8 hours (H) after the procedure, with full therapeutic doses beginning on the following day (D+1). Regular DOAC therapy was resumed within two to seven days after surgery (D+2 to D+7), tailored to individual patient needs and bleeding risk assessments. Figure S2 : **Impact of Dosage Variations on D-dimer reduction** This scatter plot illustrates the percentage of D-dimer reduction stratified by DOAC dosage levels. Categories range from 'Very Low' with Apixaban 2.5 mg once daily, 'Low' including Apixaban 2.5 mg twice daily, Rivaroxaban 10 mg once daily, and Dabigatran 75 mg twice daily, 'Intermediate' for Rivaroxaban 15 mg once daily and Dabigatran 110 mg twice daily, to 'High' for Dabigatran 150 mg twice daily, Rivaroxaban 20 mg once daily and Apixaban 5 mgr twice daily. The observed variations in D-dimer reduction across these categories may be influenced by the clinical severity of the patients' conditions, which often dictates the prescribed dosage. This indicates that those with more advanced disease may receive higher doses, which is a factor to consider when interpreting the treatment response.
