# Supplementary material for: Direct oral anticoagulants and venous malformations: literature review and retrospective study of 29 patients
Source: Res Pract Thromb Haemost. 2024 Apr 3;8(3):102400. doi: 10.1016/j.rpth.2024.102400 (PMC11087699; doi:10.1016/j.rpth.2024.102400)

# **Supplementary Appendix**

**Table S1: Direct Oral Anticoagulant therapy for venous malformations: A comprehensive review of case studies**

| **YEAR** | **REF** | **N OF PATIENTS** | **CONCLUSION** |
| --- | --- | --- | --- |
| **2013** | Randrianarisoa E (18) | 1 | **Rivaroxaban** was effective in treating recurrent deep venous thrombosis and life- threatening bleeding due to consumptive coagulopathy in an 18-year-old Caucasian boy with Klippel-Trenaunay syndrom |
| **2014** | Vandenbriele C (19) | 1 | **Rivaroxaban** was used in a patient with extensive venous malformation complicated by localized intravascular coagulopathy with recurrent and severe bleeding, with both well tolerance and improvement of fibrinogen |
| **2016** | Ardillon L (20) | 1 | A case study comparing two therapies (LMWH versus **dabigatran**) in the same individual with extensive venous malformations showed similar efficacy in controlling the consumptive coagulopathy. When used at a low dose (110 bid), its efficacy in vivo was lower than LMWH |
| **2017** | Yasumoto A (21) | 1 | **Dabigatran** was successfully used to treat a 17-year-old Japanese girl with multifocal venous malformations and diffuse intravascular coagulopathy |
| **2018** | Binet Q (22) | 3 | A retrospective case series involving 3-patients presented the success of using **dabigatran** for the control of localized intravascular coagulopathy (primary outcome: absence of thrombo-haemorrhagic events, secondary outcomes: laboratory markers of D-dimer and fibrinogen levels). The case series showed that dabigatran could be used as a safe and effective alternative to LMWH |
| **2018** | Mack (23) | 4 | **Rivaroxaban** was effective in controlling signs and symptoms of consumptive coagulopathy with no evidence of bleeding in 4 patients |
| **2019** | Janjetovic S (24) | 1 | **Apixaban** showed promising safety and efficacy in the management of diffuse intravascular coagulopathy caused by venous malformation in a young female with Klippel-Trenaunay syndrom |
| **2022** | Oo HP (25) | 1 | Low dose **rivaroxaban** was successful in controlling symptoms of chronic localized intravascular coagulopathy in a 38-year-old woman with venous malformation due to a variant in the TEK gene |
| **2022** | Van der Vleuten C (26) | 14 | 14 patients with Klippel-Trenaunay syndrom were treated with a DOAC as a follow-up treatment after serious thrombo-embolic events but also for painful superficial vein thrombosis. |
| **2023** | Liu H (27) | 19 | **Dabigatran** was efficacious in controlling consumptive coagulopathy and pain associated with venous malformations in a retrospective study of 19 patients. |

[18] Randrianarisoa E, Kopp H-G, Balletshofer BM, Jaschonek K, Kanz L, Haering H-U, et al. Management of disseminated intravascular coagulopathy with direct factor Xa inhibitor rivaroxaban in Klippel-Trénaunay syndrome. *Blood Coagul Fibrinolysis* 2013; **24**: 766–70. https://doi.org/10.1097/MBC.0b013e3283626238.

[19] Vandenbriele C, Vanassche T, Peetermans M, Verhamme P, Peerlinck K. Rivaroxaban for the treatment of consumptive coagulopathy associated with a vascular malformation. *J Thromb Thrombolysis* 2014; **38**: 121–3. https://doi.org/10.1007/s11239-013-1024-7.

[20] Ardillon L, Lambert C, Eeckhoudt S, Boon LM, Hermans C. Dabigatran etexilate versus low-molecular weight heparin to control consumptive coagulopathy secondary to diffuse venous vascular malformations. *Blood Coagul Fibrinolysis* 2016; **27**: 216–9. https://doi.org/10.1097/MBC.0000000000000412.

[21] Yasumoto A, Ishiura R, Narushima M, Yatomi Y. Successful treatment with dabigatran for consumptive coagulopathy associated with extensive vascular malformations. *Blood Coagul Fibrinolysis* 2017; **28**: 670–4. https://doi.org/10.1097/MBC.0000000000000666.

[22] Binet Q, Lambert C, Hermans C. Dabigatran etexilate in the treatment of localized intravascular coagulopathy associated with venous malformations. *Thromb Res* 2018; **168**: 114–20. https://doi.org/10.1016/j.thromres.2018.06.013.

[23] Mack JM, Richter GT, Crary SE. Effectiveness and Safety of Treatment with Direct Oral Anticoagulant Rivaroxaban in Patients with Slow-Flow Vascular Malformations: A Case Series. *Lymphat Res Biol* 2018; **16**: 278–81. https://doi.org/10.1089/lrb.2017.0029.

[24] Janjetovic S, Holstein K, Dicke C, Bokemeyer C, Langer F. Apixaban for the Treatment of Chronic Disseminated Intravascular Coagulation: A Report of Two Cases. *Hamostaseologie* 2019; **39**: 294–7. https://doi.org/10.1055/s-0038-1675386.

[25] Oo HP, Pasricha S-R, Thompson B, Winship I, Scardamaglia L. Rivaroxaban in the treatment of TEK-related venous malformation. *Australas J Dermatol* 2022; **63**: e255–8. https://doi.org/10.1111/ajd.13856.

[26] Van der Vleuten CJM, Zwerink LGJM, Klappe EM, de Jong EMGJ, Te Loo DMWM. Is there a place for prophylaxis with DOACs in Klippel-Trenaunay syndrome and other low-flow vascular malformations with intravascular coagulopathy and thromboembolic events? *Thromb Res* 2022; **213**: 30–3. https://doi.org/10.1016/j.thromres.2022.03.004.

[27] Liu H, Hu L, Yang X, Xu Z, Gu H, Chen H, et al. Dabigatran etexilate is efficacious in consumptive coagulopathy and pain associated with venous malformations. *J Vasc Surg Venous Lymphat Disord* 2023; **11**: 397-403.e1. https://doi.org/10.1016/j.jvsv.2022.09.015.

**Table S2: Overview of Variables Assessed in Venous Malformation Study**

| **CHARACTERISTICS OF STUDY POPULATION**   - Age (years) - Gender (F/M) - Size of VM’s   - Localized (<9% of BSA)   - Extensive (>9% of BSA) - Location of VM’s - Specific diagnosis - Genetic variant | - History of hemophilic arthropathy - History of vascular surgery or sclerotherapy - History of SVT, DVT or PE - History of LIC - History of postoperative haemorrhage - Previous anticoagulation (LMWH, acenocoumarol) - Reason for previous anticoagulation - Other treatment (sirolimus) |
| --- | --- |
| **DOAC**   - Type of DOAC - Dose of DOAC   “Very low”: Apixaban 2,5 1x “Low” : Apixaban 2,5 2x, Rivaroxaban 10 1x, Dabigatran 75 2x “Intermediate” : Rivaroxaban 15 1x, Dabigatran 110 2x “High” : Apixaban 5 2x, Rivaroxaban 20 1x, Dabigatran 150 2x | - Reason for treatment - TEE under DOAC - Duration of treatment (years) - DOAC interruption for intervention and pregnancy - Perioperative management of anticoagulation |
| **LAB RESULTS**   - Platelet count nadir (X/microL) - Fibrinogen level nadir (mg/dL) - Maximum level of D-dimer - D-dimer before and after DOAC - Bleeding disorder screening - Thrombophilia screening | **CLINICAL EVALUATION**   - Pain improvement - Bleeding event (and location) |

This table enumerates the variables examined in the study of patients with venous malformations. Key abbreviations include F (Female), M (Male), BSA (Body Surface Area), VMs (Venous Malformations), SVT (Superficial Vein Thrombosis), DVT (Deep Vein Thrombosis), PE (Pulmonary Embolism), LIC (Localized Intravascular Coagulopathy), LMWH (Low Molecular Weight Heparin), DOAC (Direct Oral Anticoagulant) and TEE (Thrombo-Embolic Event). The table captures a broad spectrum of patient characteristics, treatment details, laboratory results, and clinical evaluations, forming the basis for a comprehensive analysis of therapeutic outcomes and complications.

**Table S3: Retrospective Study : Patient Data and Results Overview**


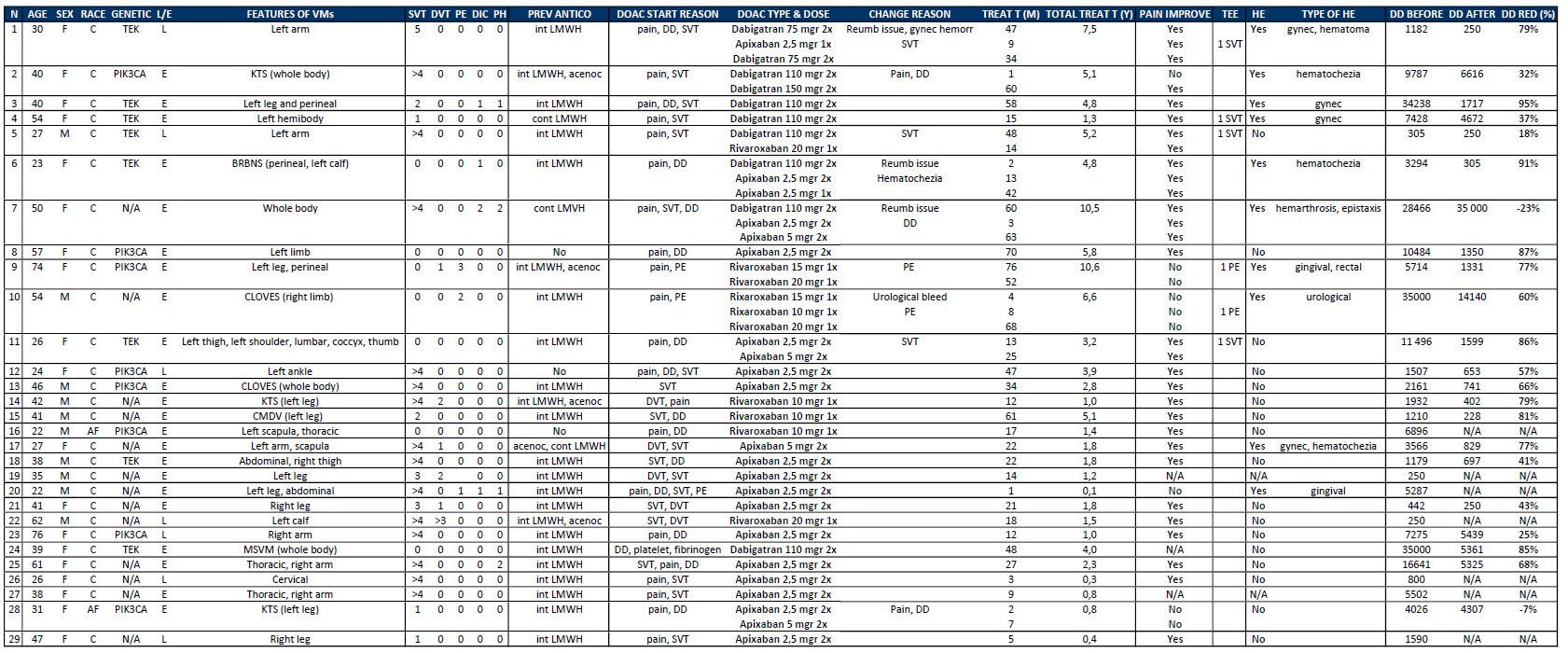

This table encapsulates the full range of data for the 29 patients involved in the study on venous malformations. Abbreviations used include L (Localized, affecting less than 9% of body surface area), E (Extensive, affecting more than 9% of body surface area), VMs (Venous Malformations), SVT (Superficial Vein Thrombosis), DVT (Deep Vein Thrombosis), PE (Pulmonary Embolism), DIC (Diffuse Intravascular Coagulation), PH (Postoperative Hemorrhage), PREV ANTICO (Previous Anticoagulation), DOAC (Direct Oral Anticoagulant), TREAT T (Treatment Time), M (Months), Y (Years), TEE (Thromboembolic Event), HE (Hemorrhagic Event), DD (D-dimer, measured in ng/Ml FEU), DD RED (D-Dimer Reduction, in percentage), F (Female), M (Male), C (Caucasian), AF (African), N/A (Data Not Available), KTS (Klippel-Trenaunay Syndrome), BRBNS (Blue Rubber Bleb Nevus Syndrome), CLOVES (Congenital Lipomatous Overgrowth, Vascular Malformations, Epidermal Nevi, Skeletal Anomalies syndrome), CMDV (Capillary Malformation with Dilated Veins), int LMWH (Intermittent Low Molecular Weight Heparin), cont LMWH (Continuous Low Molecular Weight Heparin), acenoc (acenocoumarol), and fibri (fibrinogen). The table presents a detailed overview of the patient characteristics, clinical history, treatment regimens, and outcomes.

**Table S4 : Pain Improvement by DOAC Dose Category**


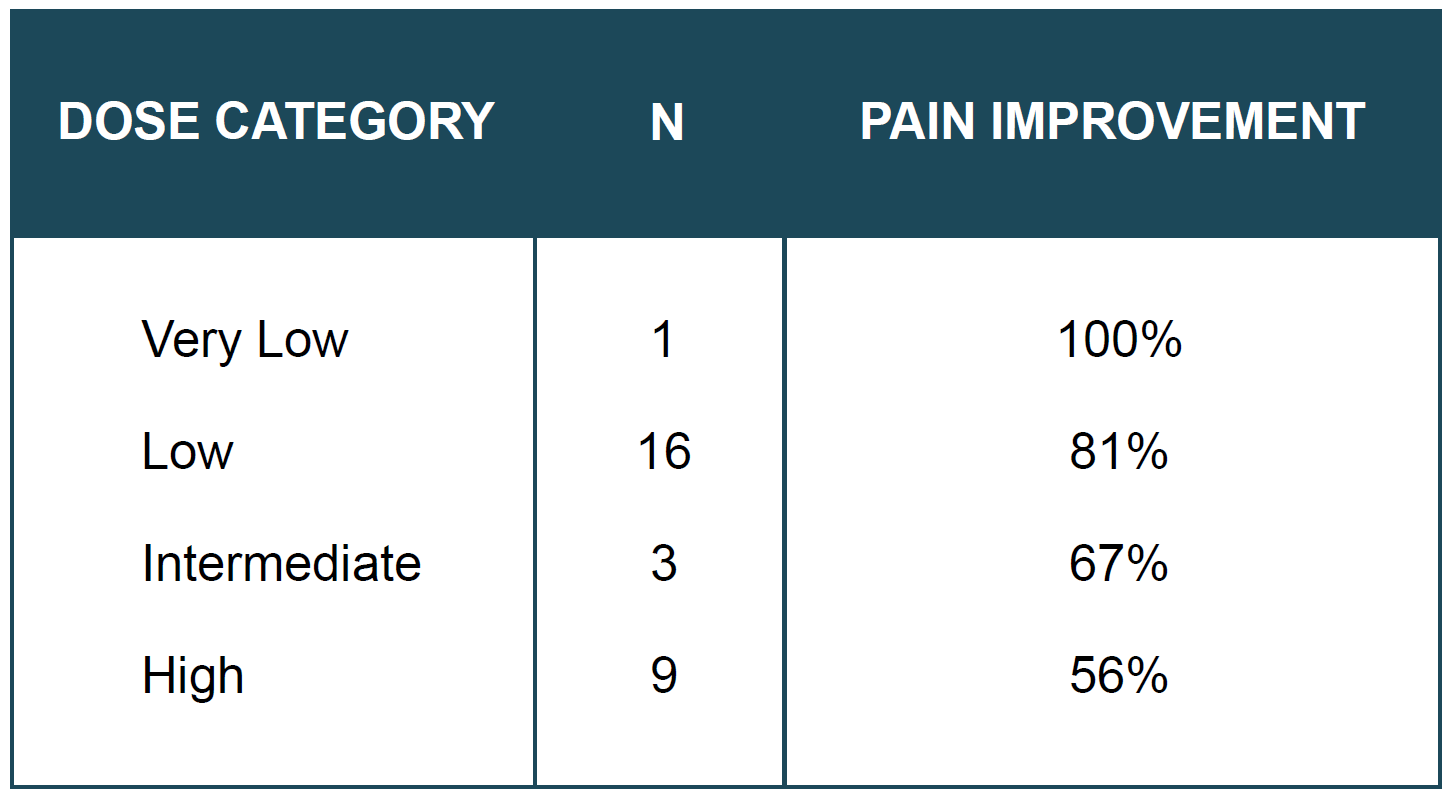


The table displays pain improvement percentages across different DOAC dosage categories. The 'Very Low Dose' category specifies patients on Apixaban 2.5 mg once daily. 'Low Dose' includes Apixaban 2.5 mg twice daily, Dabigatran 75 mg twice daily, or Rivaroxaban 10 mg once daily. 'Intermediate Dose' applies to Dabigatran 110 mg twice daily or Rivaroxaban 15 mg once daily, and 'High Dose' to Dabigatran 150 mg twice daily or Rivaroxaban 20 mg once daily. The observed pattern, where increased dosage correlates with less pain improvement, may reflect that patients prescribed higher doses often have more severe disease manifestations. These cases may inherently be less responsive to pain management, which should not be mistaken for reduced efficacy of higher DOAC dosages.

**Figure S1 : Perioperative Management of Anticoagulation in Patients on direct oral anticoagulant**


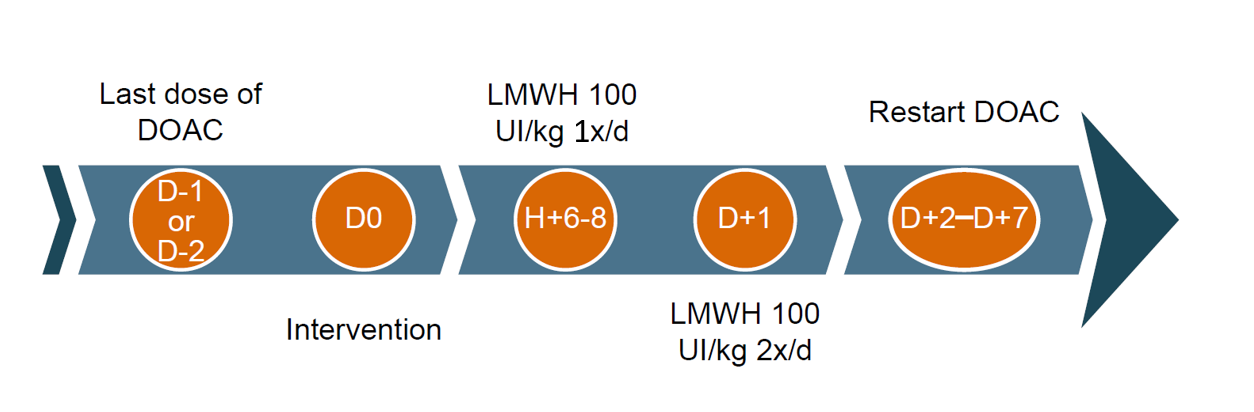


**Figure S2 : Impact of Dosage Variations on D-dimer reduction**


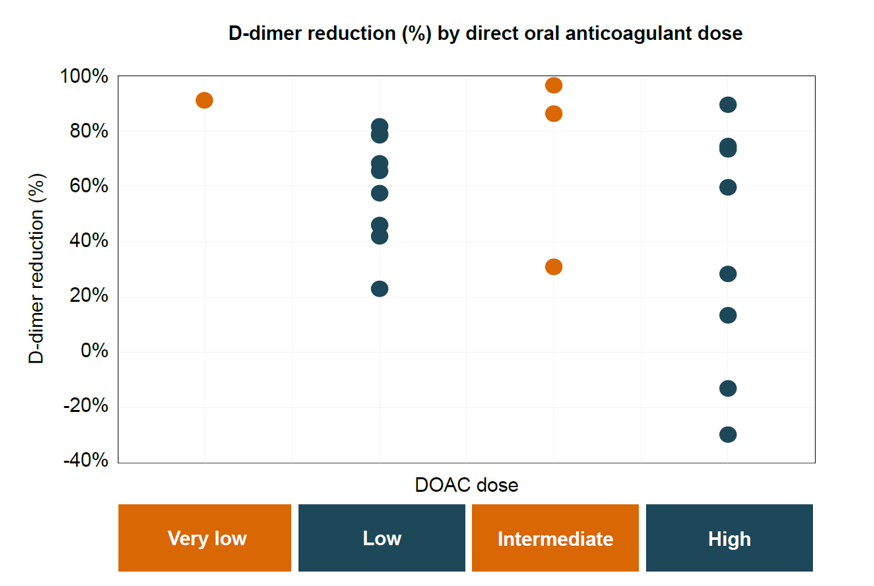

Supplement: Figure S1 — Perioperative Management of Anticoagulation in Patients on direct oral anticoagulant The timeline details anticoagulation management for patients undergoing surgery. For low-dose DOAC patients, the last dose was administered either the morning before surgery (D-1) or two mornings prior for surgeries with a high risk of bleeding (D-2). High-dose DOAC patients stopped their medication two days before surgery (D-2). Postoperatively, semi-therapeutic doses of LMWH were started 6-8 hours (H) after the procedure, with full therapeutic doses beginning on the following day (D+1). Regular DOAC therapy was resumed within two to seven days after surgery (D+2 to D+7), tailored to individual patient needs and bleeding risk assessments. Figure S2 : Impact of Dosage Variations on D-dimer reduction This scatter plot illustrates the percentage of D-dimer reduction stratified by DOAC dosage levels. Categories range from 'Very Low' with Apixaban 2.5 mg once daily, 'Low' including Apixaban 2.5 mg twice daily, Rivaroxaban 10 mg once daily, and Dabigatran 75 mg twice daily, 'Intermediate' for Rivaroxaban 15 mg once daily and Dabigatran 110 mg twice daily, to 'High' for Dabigatran 150 mg twice daily, Rivaroxaban 20 mg once daily and Apixaban 5 mgr twice daily. The observed variations in D-dimer reduction across these categories may be influenced by the clinical severity of the patients' conditions, which often dictates the prescribed dosage. This indicates that those with more advanced disease may receive higher doses, which is a factor to consider when interpreting the treatment response. [file mmc1.docx]
